# Characterization of patients with vulvar lichen sclerosus and association to vulvar carcinoma: a retrospective single center analysis

**DOI:** 10.1007/s00404-022-06848-y

**Published:** 2022-11-21

**Authors:** L. Steinkasserer, J. Hachenberg, P. Hillemanns, M. Jentschke

**Affiliations:** Department of Gynaecology and Obstetrics, Hannover Medical School, Hannover, Germany

**Keywords:** Vulvar lichen sclerosus, Vulvar intraepithelial neoplasia, Vulvar squamous cell carcinoma

## Abstract

**Purpose:**

Lichen sclerosus (LS) is a benign, cutaneous, chronic inflammatory (autoimmunological) disease. The differentiated vulvar intraepithelial neoplasia (dVIN) accounts for a precursor lesion of vulvar squamous cell carcinoma and is often associated with lichen sclerosus. Although the association between lichen sclerosus and vulvar carcinoma has long been recognized, there is a lack of evidence in literature.

**Methods:**

This retrospective study examined pseudonymized data of 499 women diagnosed with vulvar pathology between 2008 and 2020 at the Department of Gynaecology and Obstetrics of Hannover Medical School (MHH). Data were further stratified for the time of onset, location of disease, accompanying disease, HPV status and progression of disease into vulvar squamous cell carcinoma (VSCC).

**Results:**

In total, 56 patients were diagnosed with vulvar lichen sclerosus. The mean onset of disease was at 60.3 years of age. After subdividing cases of diagnosed LS into those who did not develop vulvar carcinoma in their course and those who did, the ages at onset are 52.66 ± 17.35 and 68.41 ± 10.87, respectively. The incidence of vulvar cancer in women diagnosed with lichen sclerosus was 48.2%. Twenty-five patients reported a diagnosis of VIN in their self-reported history.

**Conclusions:**

In our retrospective study, we showed a trend between vulvar lichen sclerosus and VSCC. The difference between the two age groups of patients diagnosed with lichen sclerosus who developed vulvar carcinoma and those who did not is statistically significant. Our results highlight the importance to diagnose lichen sclerosus early to ensure adequate follow-up and prevent progression to VSCC.

**Supplementary Information:**

The online version contains supplementary material available at 10.1007/s00404-022-06848-y.

## What does this study adds to the clinical work

**Table:** 

The aim of the study was to show the particular characteristics of patients with lichen sclerosus and investigating the incidence of progression into a vulvar squamous cell carcinoma. Our results highlight the importance to diagnose lichen sclerosus early to ensure adequate follow-up and prevent progression to vulvar squamous cell carcinoma.	

## Background

Lichen sclerosus (LS) is a chronic inflammatory, autoimmunological disease of unknown etiology characterized by particular dermal transformations and commonly appears as white, atrophic plaques [1]. The etiology of LS still is unknown. Apart from genetic and infectious factors, immunological abnormalities and hormonal factors are discussed [2–8]. Women with vulvar LS tend to have a slightly increased risk (2–5%) of developing vulvar squamous cell carcinoma (VSCC) [9–16]. Vulvar squamous intraepithelial lesions (SIL) include low-grade squamous intraepithelial lesions (LSIL), high-grade squamous intraepithelial lesions (HSIL), and differentiated vulvar intraepithelial neoplasia (dVIN). Whereas VIN 1 is classified as a mild dysplasia and accounts to a low-grade intraepithelial lesion, VIN 2 and VIN 3 as moderate and severe dysplasia are classified as high-grade squamous intraepithelial lesions [17]. VSCC represents a carcinoma that in general arises from a precancerous lesion, the high-grade vulvar intraepithelial neoplasia (VIN). According to the 2015 International Society for the Study of Vulvovaginal Disease (ISSVD), precancerous lesions can further be subclassified into two types. The usual VIN type (uVIN) which is induced by an infection with high-risk genotypes of human papillomavirus (HPV) and the HPV independent or differentiated VIN (dVIN) [17]. Due to its non-specific clinical characteristics, dVIN as a solitary lesion is difficult to diagnose. It often may easily be mistaken for a benign dermatosis. The underdiagnosis may be explained due to its challenging identification or due to its short intraepithelial phase [10, 11, 15, 18]. It is suggested that dVIN may develop from LS. If both pathologies occur simultaneously, the risk of developing VSCC increases [13, 19, 20]. Early detection and early adequate therapy may lead to a reduction in risk of VSCC for women diagnosed with LS [9, 21].

The true prevalence of LS is not known. Incidences range from 1 in 30 to 1 in 1000 patients [9, 22, 23]. The incidence of vulvar high-grade squamous intraepithelial lesions (HSIL) (VIN 2 and VIN 3) ranges from 2.86 to 3.26 per 100,000 women [11, 24].

In 2018, approximately 3270 women developed a malignant neoplasm of the vulva, and 957 women died from this disease in the same year in Germany [25]. VSCC represents the largest proportion of all vulvar carcinomas, accounting for more than 90% [11, 26]. As age increases, so does the incidence of vulvar cancer. In addition to VIN and vulvar LS, nicotine abuse, autoimmune diseases, a prior history of cervical cancer, and Northern European descent account as important risk factors to develop vulvar carcinoma [27, 28]. The relative 5-year overall survival rate of a malignant vulvar tumor is 73%. Most vulvar cancers are detected at the early tumor stage [25].

Although the association between LS and vulvar cancer is well known, further studies are missing to close the lack of data. Our study aimed to show the characterization of patients with vulvar lichen sclerosus and to examine correlation between vulvar LS and VSCC.

## Methods

### Patient cohort

For this study, histological findings and clinical data of patients with various vulvar pathologies, who were registered at the Department of Obstetrics and Gynecology of Hannover Medical School (MHH) from 2008 to 2020 were identified retrospectively.

Before collecting the data, the ethics committee was asked to evaluate the study (No. 9683_BO_K_2021). All methods were carried out in accordance with relevant guidelines and regulations. Informed consent was obtained from all subjects or their legal guardians under Ethical approval and consent to participate section. Data were analyzed about the exact diagnosis, time of initial diagnosis, type of treatment, symptoms and course of the disease. This was realized using the Enterprise Clinical Research Data Warehouse (ECRDW). ECRDW of MHH is a multidisciplinary platform for research-relevant issues. The data stock of this ECRDW consist of consolidated, high-quality data from heterogeneous systems of MHH. The patient population was searched for the following International Statistical Classification of Diseases codes (ICD-10): N90.4, N90.5, N90.6, N90.8, N90.9 and C51. Every report contains patient identification, all diagnoses of the patients, the exact date of diagnosis, codes of surgery and routines (“Operationen- und Prozeduren-Schlüssel”; OPS), physician’s letters, and pathological findings and histology. HPV status was performed by PCR method in our pathology department.

Furthermore, cases with possible LS were included in this study. Possible LS included cases with interface dermatitis that could fit with an early phase of LS. Cases were also classified as LS when no histology was done, but the clinical appearance indicated LS. Further cases were included where LS was diagnosed within the scope of VSCC treatment.

The therapy for patients with lichen sclerosus usually includes local therapy with cortisone ointment according to the following schedule: 6 weeks daily, 4 weeks 2–3 times a week and then permanently 1–2 times a week. The follow-up of the patients includes an annual follow-up in our dysplasia consultation with anamnesis of the symptoms, vulvoscopy and, if necessary, biopsy.

### Statistical analysis

All cases were collected in a database and evaluated using Microsoft Excel 2021 (version 16.56; Microsoft Corp., Redmond, WA, USA). Data are presented as means ± standard deviation (SD). Distribution was examined with ther Shapiro–Wilk normality test, and groups were compared with an unpaired *t* test or Mann–Whitney-*U* test as appropriate after checking for outliers. Data were analyzed with the Prism 7 software package (GraphPad Software, La Jolla, CA).

## Results

**Fig. 1:**
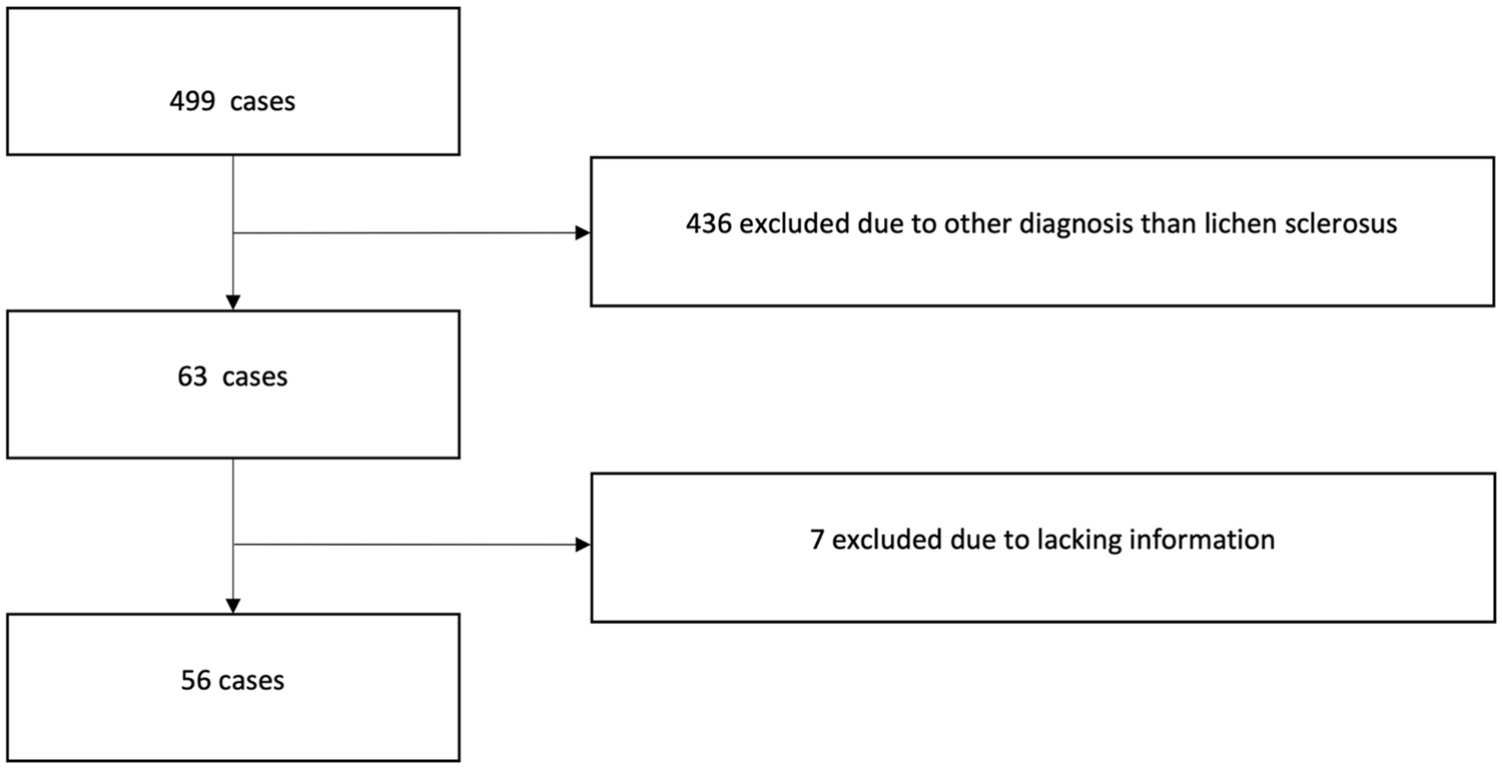
Consort diagram

A total of 499 cases with various vulvar diseases were derived from the database of the Department of Obstetrics and Gynecology of the Hannover Medical School (MHH) from 2008 to 2020. 436 cases were excluded due to another diagnosis than LS. The final study population comprised a total of 56 cases (Fig. 1).

The general characteristics of the patients with LS are shown in Table 1.

**Table 1:** Characteristics of women with lichen sclerosus

Characteristics	Median (range)	Standard deviation
Age	60.25 (16–85)	16.38

Characteristics	*N*	%
Menopausal status at the time of diagnosis		
Postmenopausal	44	78.57
Prämenopausal	12	21.43
HPV status		
Positive	6	10.72
Negative	18	32.14
Not done	32	57.14
Confirmed histology		
Yes	28	50
No	28	50
Parity		
Nullipara	12	21.43
Unipara	21	37.5
≥ 2 Para	21	37.5
Unknown	2	3.57
Nicotine abuse		
Yes	8	14.29
No	43	76.79
Ex-smoker	2	3.57
Unknown	3	5.35
Pre-existing diseases		
Arterial hypertension	24	42.86
Hypothyroidism	15	26.79
Diabetes mellitus	13	23.21
Presence of VIN		
VIN I-III	25	44.64
VIN I	5	20
VIN II	7	28
VIN II-III	1	4
VIN III	12	48

The average age for the first diagnosis of LS was 60.25 years of age (range 16–85). The majority of 78.57% developed the disease in the postmenopausal period (Fig. 1 spp).

**Fig. 2:**
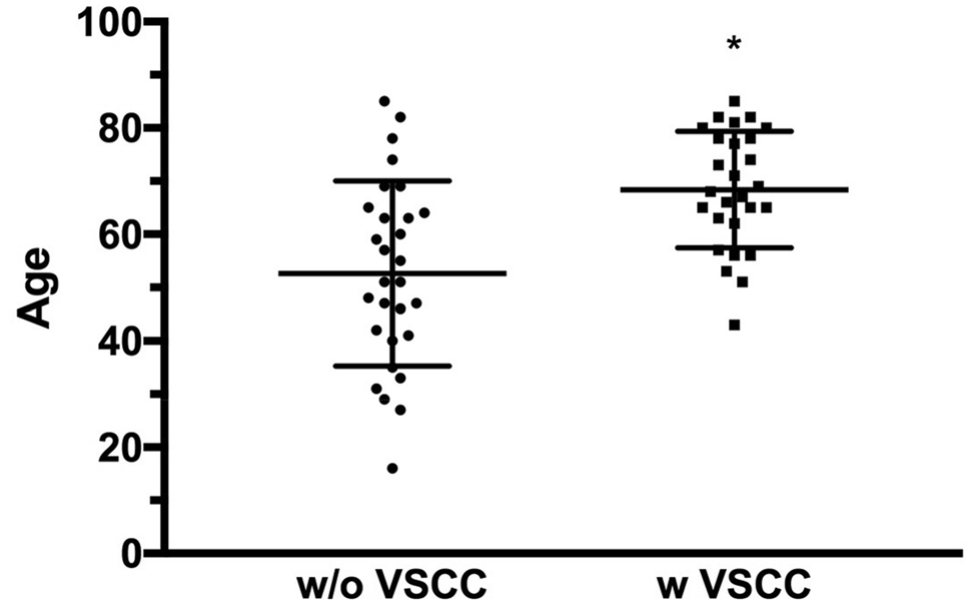
The difference between the two age groups at onset of patients diagnosed with lichen sclerosus who developed vulvar carcinoma (w VSCC: with VSCC) and those who did not (w/o VSCC: without VSCC) is statistically significant (*p* value 0.0213). The standard deviation of the age of patients with lichen sclerosus without VSCC and patients with lichen sclerosus with VSCC is 17.35 and 10.87, respectively

The median age of onset for VSCC was higher than the age at first LS diagnosis and amounts to 67.53 years of age (Fig. 2 spp). In the case of vulvar carcinoma, the majority of the cohort was postmenopausal (92.5%).

After further subdividing cases of diagnosed LS into those who did not develop vulvar carcinoma in their course and those who did, the ages at onset are 52.66 ± 17.35 and 68.41 ± 10.87 respectively (Table 2). The difference between the two age groups is statistically significant (*p* = 0.021) (Fig. 2).

**Table 2:** Age of the first diagnosis

Group	*N*	Age at first LS diagnosis	
		Mean	Range
LS	56	60.3	16–85
Without VSCC	29	52.66	16–85
With VSCC	27	68.41	43–85

Regarding other risk factors, eight patients reported positive nicotine abuse in their medical history, whereas the majority of patients stated that they did not smoke (76.8%). In two patients, the smoking status could not be determined retrospectively.

**Fig. 3:**
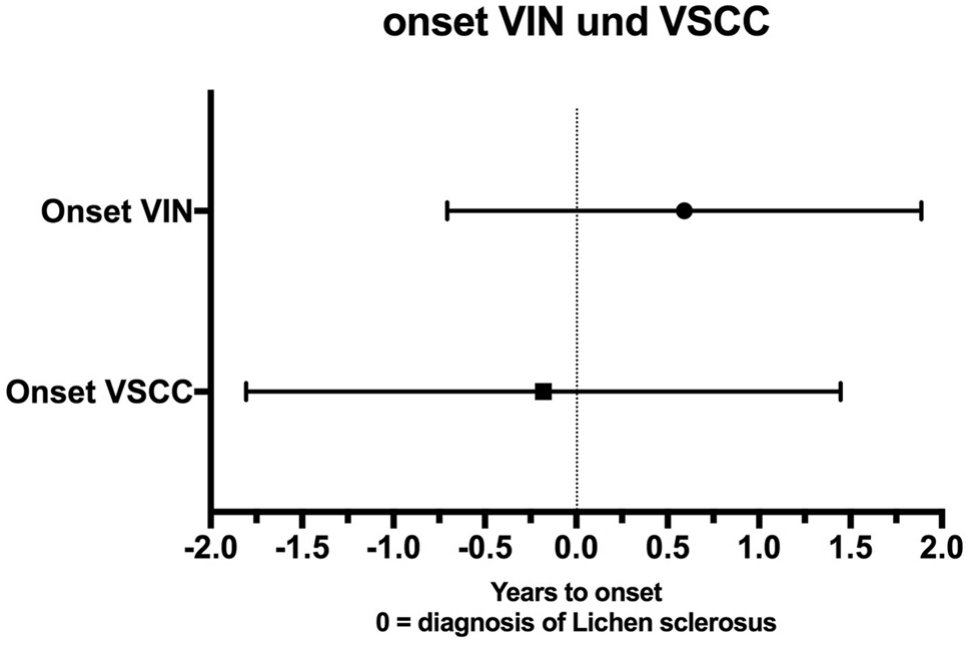
The mean time between initial diagnosis of lichen sclerosus and VIN amounts to 0.6 years. Considering the time between initial diagnosis of lichen sclerosus and further progression to vulvar carcinoma, the mean value is − 0.3 years

Twelve patients were nulliparous, 21 patients were primiparous and 21 patients delivered more than one baby. In 2 cases parity was unknown. All related diseases are shown in Fig. 3a. spp. The most common secondary diagnoses in our patient population were arterial hypertension (42.9%), hypothyroidism (26.8%), and diabetes mellitus (12.5%). Other secondary diagnoses have been divided into different groups to provide a better overview: oncological diseases, cardiological diseases, psychiatric diseases, dermatological diseases, hepatic diseases, thrombosis/embolism, arthrosis, nephrological diseases, and pulmonary disease. The diseases of the different groups are shown in Table 3 and Fig. 3 spp.

**Table 3:** Classification of secondary diagnoses

Group	Classification
Arterial hypertension	
Hypothyroidism	
Diabetes mellitus	
Oncological diseases	Colon carcinoma
	Urothelial cancer cholangiocellular carcinoma
	Thyroid carcinoma
	Colorectal carcinoma
	Breast cancer
	Cervical carcinoma
Rheumatic diseases	Psoriasis
	Arthritis
	Hyperuricaemia
	Rheuma
Cardiological diseases	Atrial fibrillation
	Congestive heart failure
	Supraventricular tachycardia
Psychiatric diseases	Dementia
	Schizophrenia
	Panic attacks
Dermatological diseases	Urticaria
	Rosacea
	Vitiligo
Hepatic diseases	Hepatic steatosis
	Hepatitis
Thrombosis/Embolism	
Nephrological diseases	Renal failure
Pulmonary disease	Bronchial asthma COPD

Fifty percentage of LS cases have been histologically confirmed. The remaining 50% were diagnosed clinically. Clinical diagnosis of lichen sclerosus was always confirmed by an experienced senior clinician. When assessing the HPV status of patients with LS 9 patients were HPV negative (32.2%), 6 patients were HPV positive (10.7%) and in 17 cases, HPV status was not done (57.1%). Twenty–five patients (44.6%) were diagnosed by VIN (20% VIN 1, 28% VIN 2, 4% VIN 2–3, 48% VIN 3).

48.2% of LS cases were associated with VSCC. The general characteristics of the patients with VSCC are shown in Table 4. Of these patients 63% raised from the precancerous lesion VIN (17.7% VIN 1, 29.4% VIN 2, 5.9% VIN 2–3, 47% VIN 3). The mean time between initial diagnosis of LS and VIN amounts to 0.6 years. Considering the time between initial diagnosis of LS and further progression to vulvar carcinoma, the mean value is − 0.3 years. Five cases of LS were diagnosed only at or after diagnosis of VSCC and therefore the time to progression amounts to − 0.3 years (Fig. 3).

**Table 4:** Characteristics of women with vulvar carcinoma

Characteristics	Median (range)	Standard deviation
Age	67.54 (34–85)	12.43

Characteristics	*N*	%
Menopausal status at the time of diagnosis		
Postmenopausal	25	92.60
Prämenopausal	2	7.40
HPV status		
Positive	1	3.70
Negative	9	33.34
Not done	17	62.96
Nicotine abuse		
Yes	5	18.52
No	18	66.67
Ex-smoker	2	7.41
Unknown	2	7.41
Presence of VIN		
VIN I-III	17	63
VIN I	3	17.64
VIN II	5	29.41
VIN II-III	1	5.89
VIN III	8	47.06

In 18 patients who developed vulvar carcinoma in course, both LS and VIN were confirmed (66.6%). Regarding the HPV status, 33.4% of VSCC were negative, and in 63% of all cases, HPV status was not done. Only one patient with VSCC was HPV positive.

Five of the patients with vulvar carcinoma were smokers while nicotine status was unknown in two cases. Interestingly, more than half of the patients (66.6%) reported not to smoke (Fig. 4 spp).

Comparing the secondary diagnoses of patients who developed VSCC against those who did not, significantly more women showed hypertension (17 and 7, retrospectively) but half as little suffered from hypothyroidism (5 and 10, retrospectively). Patients who did not develop VSCC are more often healthy. The two cohorts did not differ in any other secondary diagnoses (Fig. 3b spp). In terms of symptomatology, the two cohorts differed only for dyspareunia and burning (Fig. 5 spp).

## Discussion

Detection of LS is of utmost importance to ensure accurate control of the disease and prevention of progression to vulvar neoplasia. To our knowledge, this is the first study in Germany describing particular characteristics of patients with LS and investigating the incidence of progression into a VSCC. In this retrospective study, we were able to show a trend between vulvar lichen sclerosus and VSCC. Furthermore, we showed the different relevant characteristics of LS and VSCC. Theoretically, the detection of LS can represent a significant advantage for the affected patients to prevent progression to VSCC, as they are more amenable to follow-up examinations. Even if progression cannot be prevented, the earlier possible detection of a malignant change would enable a faster appropriate therapy.

The strength of this study is that we described and investigated a unique cohort with specific characteristics. The most common secondary diagnoses in our patient population were arterial hypertension, hypothyroidism, and diabetes mellitus. There are studies which showed similar results in reporting thyroid diseases, hypertension, overweight, increasing age and anorectal fissures as significantly associated with vulvar lichen sclerosus [29, 30]. The association between thyroid diseases and lichen sclerosus could also be shown in other publications [21, 31–33]. The studies that investigate the characteristics of the patient collective with LS are rare. More studies are urgently needed to describe the population of a rare disease and to increase the probability of being diagnosed and treated when affected.

The analysis was limited by the fact that there is no general ICD-10 code for genital LS. LS is generally coded as L90.0. However, female genital LS is explicitly excluded here, and reference is made to N90.4. Therefore, to analyze patients with LS, different ICD-10 codes had to be considered. Due to the inaccuracy of the coding possibility of this disease, it cannot be guaranteed that physicians used exactly those codes to describe LS which were analyzed here. Another weakness of this study was that 21 cases of LS were not detected until VSCC diagnosis. This could be an indication that some cases of LS remained undetected until a VSCC was diagnosed. These cases were described first or even after when patients came for therapy of VSCC. This explains the unusually high incidence of VSCC in our cohort of LS patients. Therefore, adequate diagnosis of LS seems to be a common problem.

A study published by Bleeker et al. investigated 3,038 women with LS diagnosed between 1991 and 2011 [10]. As important risk factors for vulvar cancer development, they named a concurrent VIN and age over 70 years when LS was diagnosed. In our study, we were able to describe the concomitant presence of VIN and advanced age in cases developing VSCC. Regarding the mean age of first LS diagnosis, our results were similar to the results of Bleeker et al. (60.3 in our study compared with 59.8 in the study by Bleeker et al.).

In our study, 48.2% of LS cases were associated with VSCC. Other studies showed a much lower percentage of cases where LS progressed to vulvar cancer [10, 11, 20]. This rather high progression rate of LS into vulvar neoplasia is certainly not least due to the fact that we included 21 cases in our study in which LS was only discovered after the diagnosis of vulvar carcinoma was confirmed. The late diagnosis of LS, which in the worst case is revealed together with a malignancy, can be explained by the fact that the clinical picture shows asymptomatic courses and the patients only consult a doctor when the symptoms are associated with the neoplasia appear. Furthermore, there are still many elder women who do not visit their gynecologists at all any more or who are ashamed to talk about typical symptoms associated with LS. This leads to a potential underdiagnosis of LS cases. This assumption is underlined by our finding that women with LS-associated VSCC are significantly older than women with LS without VSCC (68 vs. 53 years). Nevertheless, it is highly important to include these cases in the analysis, due to the present association between LS and VSCC. The loss of patients without a histologically confirmed diagnosis and patients with a simultaneous diagnosis of LS and VSCC would result in a significant bias, especially concerning some characteristic features like age of the first onset of the patient collective.

The statistical significance between the age groups of patients diagnosed with lichen sclerosus who developed vulvar carcinoma and those who did not shows that early diagnosis and adequate follow-up could prevent the progression of lichen sclerosus into carcinoma.

Van de Nieuwenhof et al. showed a significantly higher occurrence of dVIN in contrast to uVIN in patients that were later diagnosed with vulvar carcinomas. The time for progression into a VSCC was also significantly shorter in patients with a dVIN compared to patients with a diagnosed uVIN [34]. In our study, it was not possible to retrospectively type the various VIN diagnoses in patients with LS. Nevertheless, retrospective analysis of HPV status was possible in 43% of LS and 30% of VSCC cases. Even if the HPV status is not routinely performed for either LS or VSCC, there are still significantly more cases with HPV negative status than HPV positive.

According to the Evidence-based (S3) Guideline on (anogenital) LS, biopsy for histological confirmation of LS is only necessary when the clinical diagnosis is uncertain, when first-line therapy is not effective, or when malignancy is suspected [35]. Accordingly, our data included cases where LS was diagnosed clinically and no corresponding histology was present.

Molecular alterations in vulva carcinoma based on lichen sclerosus are rarely described. A study published by Rotondo et al. showed that hypermethylation-induced RARß down-expression is associated with the progression of lichen sclerosus in vulvar carcinoma. Further there were able to show, that with degree of methylation of RARß promoter the malignancy of VSCC based on lichen sclerosus increased [36]. However, it can be concluded that not only early detection, but also molecular alterations are determining factors for the progression of lichen sclerosus into VSCC.

In this study, we described the group of patients with LS in more detail. Even not statistically significant LS trended to progress into a VSCC. Especially older women with undetected LS have a risk of developing VSCC. Nevertheless, further research is required.

## Conclusions

In our retrospective study we analyzed 56 patients with lichen sclerosus with regard to exact diagnosis, time of initial diagnosis, type of treatment, symptoms and course of the disease.

We were able to show a trend between vulvar lichen sclerosus and VSCC.

Furthermore, we showed the different relevant characteristics of LS and VSCC. Theoretically, the detection of LS can represent a significant advantage for the affected patients to prevent progression to VSCC, as they are more amenable to follow-up examinations.

The difference between the two age groups of patients diagnosed with lichen sclerosus who developed vulvar carcinoma and those who did not is statistically significant. This means that that early diagnosis and adequate follow-up could prevent the progression of lichen sclerosus into carcinoma. Even if progression cannot be prevented, the earlier possible detection of a malignant change would enable a faster appropriate therapy.

To our knowledge this is the first study in Germany describing particular characteristics of patients with LS and investigating the incidence of progression into a VSCC.

Our results highlight the importance to diagnose lichen sclerosus early to ensure adequate follow-up and prevent progression to VSCC.

## Supplementary Information

Below is the link to the electronic supplementary material.

Supplementary file1 (JPG 24 KB)

Supplementary file2 (JPG 23 KB)

Supplementary file3 (JPG 260 KB)

Supplementary file4 (JPG 110 KB)

Supplementary file5 (JPG 125 KB)

## Data Availability

Raw data were generated at Department of Obstetrics and Gynecology of Hannover Medical School (MHH). The datasets used and analyzed during the current study are available from the corresponding author on reasonable request.

## Abbreviations

LS: Lichen sclerosus
HPV: Human papillomavirus
VIN: Vulvar intraepithelial neoplasia
dVIN: Differentiated vulvar intraepithelial neoplasia
uVIN: Usual VIN type
MHH: Hannover medical school
VSCC: Vulvar squamous cell carcinoma
ISSVD: International society for the study of vulvovaginal disease
HSIL: High-grade squamous intraepithelial lesions
ECRDW: Enterprise clinical research data warehouse
ICD-10: International statistical classification of diseases codes
OPS: Operationen- und Prozeduren-Schlüssel
