# Supplementary figures and images for: Characterization of patients with vulvar lichen sclerosus and association to vulvar carcinoma: a retrospective single center analysis
Source: Arch Gynecol Obstet. 2022 Nov 21;307(6):1921–8. doi: 10.1007/s00404-022-06848-y (PMC10147807; doi:10.1007/s00404-022-06848-y)

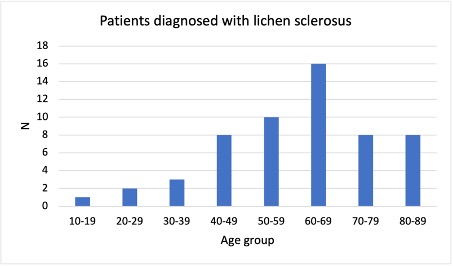

Supplement: Supplementary file 1 — Supplementary file1 (JPG 24 KB) [file 404_2022_6848_MOESM1_ESM.jpg]

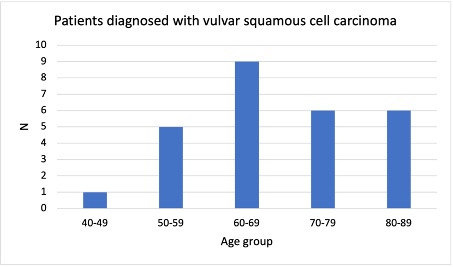

Supplement: Supplementary file 2 — Supplementary file2 (JPG 23 KB) [file 404_2022_6848_MOESM2_ESM.jpg]

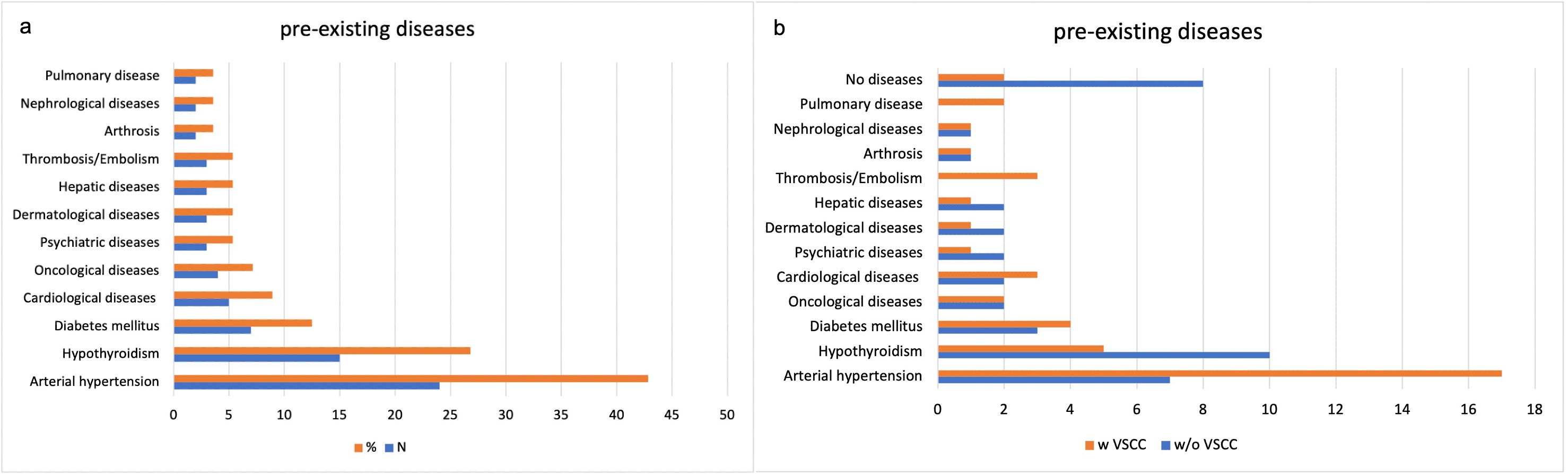

Supplement: Supplementary file 3 — Supplementary file3 (JPG 260 KB) [file 404_2022_6848_MOESM3_ESM.jpg]

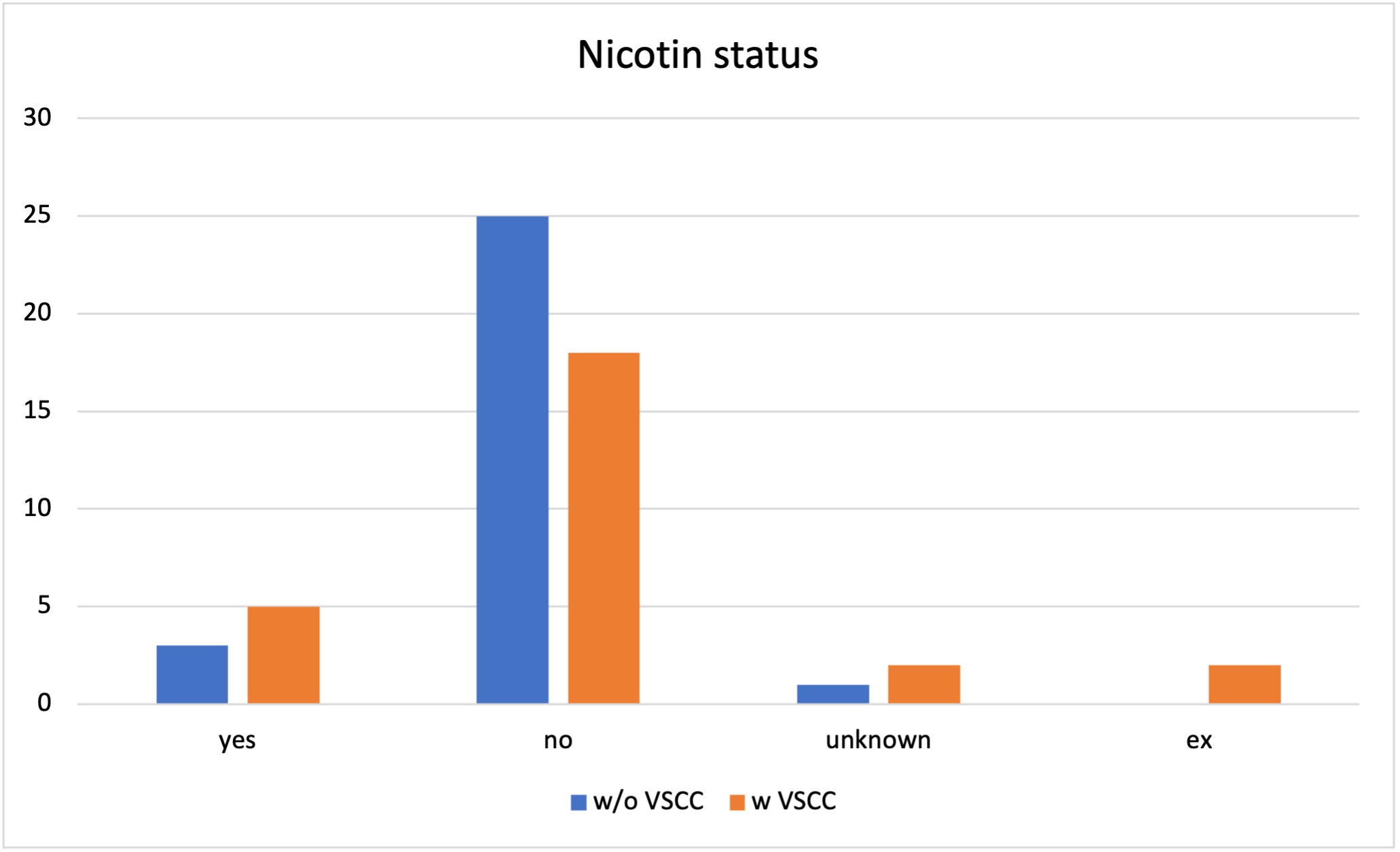

Supplement: Supplementary file 4 — Supplementary file4 (JPG 110 KB) [file 404_2022_6848_MOESM4_ESM.jpg]

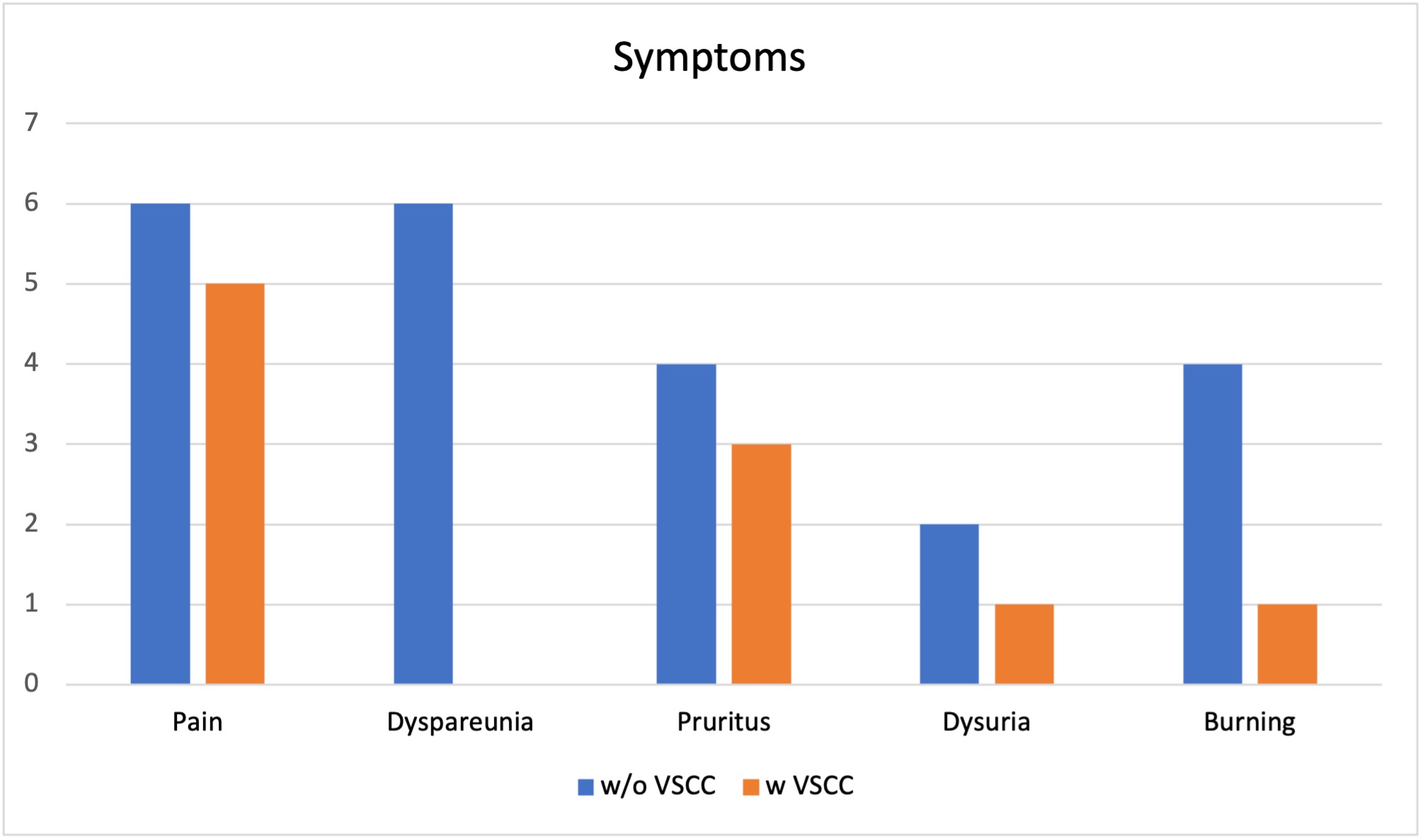

Supplement: Supplementary file 5 — Supplementary file5 (JPG 125 KB) [file 404_2022_6848_MOESM5_ESM.jpg]
